# Harnessing IL-2 for immunotherapy against cancer and chronic infection: a historical perspective and emerging trends

**DOI:** 10.1038/s12276-024-01301-3

**Published:** 2024-09-02

**Authors:** Se Jin Im, Kyungmin Lee, Sang-Jun Ha

**Affiliations:** 1 Department of Immunology, Sungkyunkwan University School of Medicine, Suwon, Korea; 2 Department of Biochemistry, College of Life Science & Biotechnology, Yonsei University, Seoul, Korea

**Keywords:** Interleukins, Translational research

## Abstract

IL-2 therapy, which enhances the function of CD8 + T cells, was initially employed as the cornerstone of immunotherapy against cancer. However, the impact of this therapy extends beyond CD8 + T cells to cells expressing IL-2R, such as endothelial cells and regulatory T cells (Tregs), resulting in various side effects. Consequently, IL-2 therapy has taken a step back from the forefront of treatment. Immune checkpoint inhibitors (ICIs), such as anti-PD-1/PD-L1 antibodies and CTLA-4 antibodies, are used because of their durable therapeutic responses and the reduced incidence of side effects. Nevertheless, only a small fraction of cancer patients respond to ICIs, and research on IL-2 as a combination treatment to improve the efficacy of these ICIs is ongoing. To mitigate side effects, efforts have focused on developing IL-2 variants that do not strongly bind to cells expressing IL-2Rα and favor signaling through IL-2Rβγ. However, recent studies have suggested that, in the context of persistent antigen stimulation models, effective stimulation of antigen-specific exhausted CD8 + T cells in combination with PD-1 inhibitors requires either 1) binding to IL-2Rα or 2) delivery via a fusion with PD-1. This review explores the historical context of IL-2 as an immunotherapeutic agent and discusses future directions for its use in cancer immunotherapy.

## Enhancing cancer immunotherapy: evolution of IL-2 therapy

Interleukin-2 is crucial for combating cancer, but its use is restricted due to severe side effects. This article reviews the development of IL-2 therapy and how it affects CD8 T cells—important immune cells that can become dysfunctional or “exhausted.” To reduce the unwanted effects, researchers have been trying to create a safer version of IL-2 that avoids targeting certain immune and body cells that contribute to these side effects. Recent studies, however, indicate that using the original form of IL-2 or specifically targeting exhausted CD8 T cells with a marker called PD-1 could be more effective in fighting tumors. Additionally, combining IL-2 therapy with PD-1 blockade can enhance T cells response to cancer. These findings suggest a promising future for personalized and effective cancer treatments. This summary was initially drafted using artificial intelligence, then revised and fact-checked by the author.

## Introduction

Interleukin-2 (IL-2), a potent T-cell-stimulating cytokine, was the first U.S. Food and Drug Administration (FDA)-approved immunotherapeutic with considerable treatment effects in metastatic melanoma and renal cell carcinoma (RCC) patients^1–3^. However, the need for high doses and frequent administration of IL-2 to induce therapeutic effects has led to severe side and off-target effects. Recognizing that these issues stem primarily from IL-2Rα-mediated signals, numerous studies have shifted their focus toward developing strategies that avoid binding with IL-2Rα^4–6^. Amidst this exploration, the field of ICIs has emerged as a novel avenue in cancer immunotherapy^7^. Antibodies (Abs), such as anti-programmed cell death-1 (PD-1)/programmed cell death ligand-1 (PD-L1) and anti-cytotoxic T-lymphocyte-associated protein 4 (CTLA-4), which have received FDA approval, are steadily expanding their indications due to sustained efficacy and minimal side effects. Nevertheless, with therapeutic effects observed in only 20–30% of patients depending on the type of cancer, ongoing research is investigating various therapies, including combination therapy, to improve outcomes. Interestingly, PD-1 has been implicated in suppressing IL-2 production by exhausted CD8 T cells and negatively regulating common γ chain cytokine receptor signals^8–10^. Conversely, PD-1 blockade is proposed to restore IL-2 production and CD8 + T-cell proliferation, and its therapeutic efficacy hinges on IL-2-mediated signaling^11^. This finding underscores the critical role of the IL-2/IL-2 receptor (IL-2R) axis in PD-1-mediated therapy. Many studies on engineering IL-2 as a combined therapy with PD-1-mediated immunotherapy have been conducted. In this review, considering the heterogeneity of exhausted CD8 + T cells, we first explored which cells exhibit responsiveness to IL-2-based therapies and how they differentiate posttreatment. Then, we summarize the development of IL-2-based therapies based on IL-2 biology and discuss the latest trends.

## Heterogeneity in CD8+T-cell exhaustion and implications for immunotherapy

### Heterogeneous differentiation of exhausted CD8+T cells and their characteristics

**Fig. 1:**
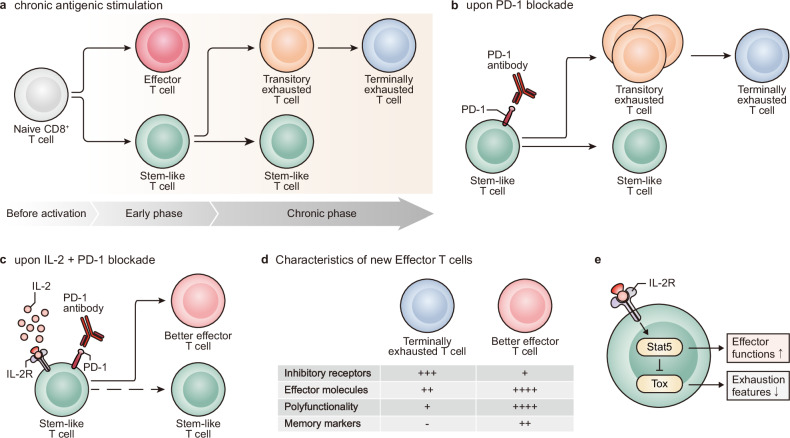
Importance of stem-like T cells and their differentiation in response to PD-1- and PD-1/IL-2-directed immunotherapies. **a** When naive T cells encounter a high antigen load, they differentiate into stem-like T cells and 1st effector cells. While 1st effector cells exhibit strong cytolytic capabilities, they eventually disappear through activation-induced cell death. In contrast, stem-like T cells sustain themselves through self-renewal and maintain antigen-specific T-cell responses by generating a transitory population and terminally exhausted CD8 T cells in response to antigenic stimulation. **b** Upon PD-1 blockade, only stem-like T cells proliferate and give rise to a substantial number of transitory cells with high cytolytic activity, thereby mediating antiviral and antitumor responses. **c** Combination therapy involving anti-PD-1 antibodies and IL-2 treatment also results in the exclusive proliferation of stem-like T cells. In contrast to the canonical differentiation pathway of exhausted CD8 T cells, stem-like T cells generate better effectors. **d** The better effectors generated by the combination therapy of IL-2 and PD-1 blockade exhibited effector-like features compared to terminally exhausted CD8 + T cells. These include decreased expression of inhibitory receptors (PD-1 and Tim-3) and increased expression of effector molecules (granzymes and perforin), inflammatory cytokines (IFNγ, TNFα, and IL-2), and memory markers (IL-7R and Lef1). **e** IL-2R-mediated signals activate STAT5, which enhances the effector functions of exhausted CD8 + T cells and antagonizes Tox, resulting in mitigated exhaustion.

T-cell exhaustion has been defined as a progressive loss of function of CD8 + T cells, including proliferative potential, cytolytic activity, and cytokine production, in a hierarchical manner due to persistent antigenic stimulation in models of chronic viral infection and cancers^12–14^. Another representative feature of exhausted CD8 T cells is the expression of inhibitory receptors, most notably PD-1^15^, whose expression increases as exhaustion progresses. We and others have recently reported the heterogeneity of exhausted CD8 + T cells in these models (Fig. 1a)^16–21^. Although a discussion about unifying the nomenclature is required, heterogeneity within exhausted CD8 + T cells is defined into a TCF1^+^ stem-like (or progenitor) subset and a Tim-3^+^ terminally differentiated subset. Based on the expression of Ki-67, CX3CR1, and CD101, Tim-3^+^ cells can be further divided into a Ki-67^+^CX3CR1^+^CD101^-^ transitory (or proliferating) population with the strongest cytolytic activity among subsets and a CX3CR1^-^CD101^+^ terminally exhausted population^22,23^.

According to the results obtained from a model of chronic lymphocytic choriomeningitis virus (LCMV) infection, naive CD8 T cells differentiate into stem-like CD8 T cells and effector cells, which possess strong functional activity, and initial high virus titers are required for the induction of stem-like CD8 T cells (Fig. 1a)^24^. In addition to TCF1, Bcl6, Bach2, Myb, and other transcription factors, which are associated with the generation of memory CD8 + T cells and follicular T helper cells, play pivotal roles in the generation of stem-like CD8 + T cells^25–28^. Tox has also been reported to be an important regulator of T-cell exhaustion, and Tox deficiency results in defects in the stem-like CD8 + T-cell subset and a failure to maintain exhausted CD8 + T cells^29–31^. Importantly, stem-like CD8 T cells act as a resource to help maintain antigen-specific CD8 T cells by sustaining their population through slow self-renewal and the continual production of Tim-3^+^ progeny cells. This cellular feature of the stem-like CD8 T-cell subset is quite similar to that of memory CD8 T cells, but they are still dysfunctional compared to memory CD8 T cells corresponding to their PD-1 expression. Antigenic stimulation initiates the differentiation of the stem-like subset into the transitory population, eventually ending with the terminally exhausted CD8 + T-cell subset, which is accompanied by proliferation and the acquisition of Blimp1 expression^22^. Interestingly, Tim-3^+^ effector cells derived from naive CD8 + T cells are quite functional, similar to effector cells generated by acute viral infection, while Tim-3^+^ terminally differentiated cells derived from the stem-like subset are functionally exhausted, although they express cytotoxic molecules, including granzymes and perforin^24^. This differentiation program has also been applied to other disease models with persistent antigenic stimulation, such as models of mouse and human tumors, autoimmune diseases^32–34^, and recently, graft-versus-host diseases^35^.

Different subsets of exhausted CD8 T cells show distinct localization patterns in chronic viral infection and cancer. In chronically infected mice, while Tim-3^+^ terminally differentiated T cells are present in both lymphoid and nonlymphoid organs, TCF1^+^ stem-like CD8 + T cells are mainly localized in lymphoid tissues such as the spleen, lymph nodes, and bone marrow^16^. Furthermore, TCF1^+^ stem-like CD8 + T cells preferentially reside in the T-cell zone of the spleen, whereas Tim-3^+^ terminally differentiated CD8 + T cells are concentrated in the red pulp. Similarly, in non-small cell lung cancer and head and neck squamous cell carcinoma (HNSCC) patients, TCF1^+^ stem-like CD8 + T cells are most likely located in tertiary lymphoid structures (TLSs), which are organized aggregates of immune cells that form within nonlymphoid tissues, not in the tumor parenchyma^21,36^. In contrast, Tim-3^+^ terminally differentiated CD8 + T cells infiltrate the tumor parenchyma and reside proximal to tumor cells. Considering that the red pulp is the major site of chronic LCMV infection and that few infected cells exist in the T-cell zone^16,36^, these results suggest that stem-like T cells are devoid of antigens, while terminally differentiated T cells interact with infected cells or tumor cells and exhibit cell-killing activity, although their cytolytic function is likely inhibited by the PD-1/PD-L1 interaction^37^. In addition to the avoidance of antigens, the preferential localization of TCF1^+^ stem-like T cells in lymphoid organs and TLSs is also associated with their maintenance. In both chronic viral infection and cancer, the stem-like CD8 T-cell subset highly expresses Xcl1, which is a ligand for XCR1^16,21,22,36^. The chemokine receptor XCR1 is exclusively expressed on CD8α^+^ lymphoid dendritic cells (DCs), called cDC1s^38^, suggesting a potential interaction between the stem-like CD8 + T-cell subset and cDC1s. Consistent with this speculation, cDC1s provide a physical niche for maintaining the population of the stem-like subset and their quiescence in chronically LCMV-infected mice^39^. Similarly, in human kidney cancer patients, stem-like CD8 + T cells reside near MHC-II-expressing cells, and the population of stem-like CD8 + MHC-II complex could suggest a clinical benefit of immunotherapy^40^. Additionally, a recent study showed that mregDCs and CXCR13^+^ T helper cells provide a niche for the differentiation of stem-like CD8 T cells following PD-1 blockade^41^. Despite the differential localization of CD8 + T-cell subsets, exhausted CD8 + T cells commonly exhibit limited circulation in both chronic viral infections and cancers. Parabiosis experiments revealed impaired migration of virus-specific CD8 + T cells between chronically infected parabionts^42^. Only a small fraction of exhausted CD8 + T cells could circulate in chronically infected mice, and the circulating cells exhibited the phenotype of CD101^-^Tim-3^+^ transitory cells. Similarly, few tumor-specific CD8 + T cells were observed in the blood of HNSCC patients, in contrast to the considerable population of tetramer^+^PD-1^hi^ CD8 + T cells in primary tumors and metastatic lymph nodes^21^.

### Stem-like CD8 T cells in antigenic stimulation and therapeutic responses

One of the important features of TCF1^+^ stem-like CD8 T cells is their exclusive proliferative potential upon antigenic stimulation. In a model of chronic LCMV infection, the sorted stem-like T-cell subset and terminally differentiated T-cell subset were transferred into infection-matched recipient mice, and only the stem-like T-cell subset could proliferate and differentiate into terminally differentiated cells^16–18^. Additionally, when the sorted stem-like CD8 T-cell subset encountered new viral challenge in naive recipient mice, it robustly proliferated and generated a large number of Tim-3^+^ progeny cells, unlike the terminally differentiated T-cell subset, which showed poor expansion. Similar proliferative attributes were observed in the two CD8 + T-cell subsets isolated from tumor-bearing mice and cancer patients upon neoantigen stimulation. For example, CD28^+^Tim-3^-^ PD-1^+^ stem-like CD8 + T cells isolated from kidney cancer patients, which contain TCF1+ cells, exhibited improved proliferation and differentiated into Tim-3^+^PD-1^+^ cells upon ex vivo anti-CD3/anti-CD28 stimulation, while the sorted Tim-3^+^PD-1^+^ CD8 + T cells showed limited proliferation^40^. Additionally, when two sorted CD8 + T-cell subsets isolated from human papilloma virus (HPV)-positive HNSCC patients were cultured with HPV peptide-loaded autologous peripheral blood mononuclear cells, only CD28^+^Tim-3^−^ PD-1^+^ CD8 + T cells could proliferate and generate Tim-3^+^ PD-1^+^ progeny cells^21^. In murine tumor models, when the two sorted CD8 T-cell subsets are exposed to cognate antigens in vivo in a type of tumor or viral infection, only the sorted stem-like CD8 T-cell subset could proliferate and generate progeny cells^20,36^. Taken together, these results suggest that only the stem-like CD8 T-cell subset possesses proliferative potential upon antigenic stimulation, and upon proliferation, this subset could differentiate into Tim-3^+^ terminally differentiated progeny cells (Fig. 1a).

In addition to its exclusive proliferative potential upon antigenic stimulation, the stem-like CD8 T-cell subset exhibited robust proliferation in response to PD-1 blockade in preclinical models and clinical models. Treatment with anti-PD-1 or anti-PD-L1 Abs significantly augmented the CX3CR1^+^Tim-3^+^ proliferating transitory population in chronically LCMV-infected mice^22^ (Fig. 1b). Importantly, the augmented transitory population was mainly derived from the stem-like T-cell subset^16,22^, in addition to compensatory self-renewal^43^. In the same manner, compared to the responsiveness of stem-like and terminally differentiated CD8 T-cell subsets isolated from tumor-bearing mice to PD-1 blockade in vivo, while the population of the terminally differentiated CD8 T-cell subset was not altered, stem-like CD8 T cells largely proliferated and were converted into terminally differentiated CD8 T cells^20^. Consistent with this observation, responders to PD-1 blockade in clinical studies indicated a greater frequency of TCF1^+^ cells among CD8 + T cells^44–46^. Accordingly, the frequency of TCF1^+^ CD8 + T cells could be used as a predictive marker to predict the responsiveness of cancer patients to ICIs.

Similar results were observed for the responsiveness to IL-2 and IL-2 plus anti-PD-1/PD-L1 combination therapy. One decade ago, the addition of IL-2 treatment to PD-1 blockade agents was shown to be highly effective at increasing virus-specific CD8 + T-cell responses, leading to a significant decrease in virus titers^47^. According to a recent study, despite the strong immune-stimulating activity of combination therapy, terminally differentiated CD8 + T cells were still unresponsive, while stem-like CD8 + T cells markedly expanded by more than 500-fold^48^ (Fig. 1c). Similarly, Deak et al. reported that the stem-like CD8 T-cell subset could exclusively proliferate after treatment with an anti-PD-1 Abs/IL-2 variant fusion protein (PD1-IL-2v) in a model of chronic LCMV infection^49^. These results suggest that the stem-like CD8 T-cell subset is a major target for immunotherapies, including PD-1 blockade combined with IL-2 treatment and the PD1-IL-2v fusion protein.

### Reshaped better effectors after IL-2-mediated immunotherapies

As we explained previously, the differentiation of exhausted CD8 T cells started from the stem-like T-cell subset, progressed into the transitory population, and ended in the terminally exhausted CD8 T-cell subset (Fig. 1a). PD-1 blockade accelerated the differentiation of stem-like T cells to a transitory population owning high cytolytic activity, which mediated the antiviral and antitumor activity^20,22^ (Fig. 1b). However, after the cessation of treatment with anti-PD-1/PD-L1 Abs, the transitory population eventually differentiated into terminally exhausted T cells, and the differentiation program returned to a steady state^50^. These results were consistent with the lack of significant changes in transcriptomic and epigenetic profiles after PD-1 blockade^51^, suggesting that the plasticity of stem-like CD8 T cells is very limited.

However, single-cell RNA sequencing data from current studies revealed that IL-2-mediated immunotherapies generated a previously unidentified new subset, called “better effectors”, because this subset had strong cytolytic activity compared to the previously identified exhausted CD8 T-cell subset^48,49,52–54^ (Fig. 1d). This unique subset exhibited a PD-1^+^Tim-3^-^GzmB^+^TCF1^low/-^ phenotype. Overall, this novel better effector subset is characterized by increased expression of genes encoding cytotoxic molecules (*Gzmb* and *Prf1*), adhesion molecules (*S1pr1*, *Itgb1, Cd44*, and *Ly6c2*), and cytokine and chemokine receptors (*Cxcr3*, *Il18ra*, *Il18rap*, and *Ifngr1*). Importantly, the expression of memory-associated genes such as *Il7r* and *Lef1* was also upregulated upon administration of IL-2-mediated immunotherapy. Considering the role of IL-7R in the generation and maintenance of memory CD8 T cells, increased expression of IL-7R on better effectors suggests that the addition of IL-7 to combination therapy or PD1-IL-2v treatment could further enhance the therapeutic efficacy of these agents against chronic viral infection and cancers. In addition to the augmented expression of effector molecules (granzymes and perforin), antigen-specific CD8 + T cells generated after IL-2-mediated therapies become polyfunctional and produce multiple cytokines, including IFNγ, TNFα, and IL-2. In contrast, they showed lower expression of inhibitory receptors (*Pdcd1*, *Havcr2*, *Lag3*, *Tigit*, *Cd160*, and *Cd244*) in addition to the *Tox* transcription factor, which is known as one of the major regulators of exhausted CD8 + T cells. One of the interesting features of better effectors is their high expression of IL-18Rα (also known as CD218a). This high expression of IL-18Rα on better effectors allows them to produce IFNγ in response to IL-12 and IL-18 in the absence of cognate antigenic stimulation. This event also occurs in human and murine bystander CD8 and CD4 T cells stimulated with IL-15^55,56^. Given that IL-15 shares IL-2Rβγ with IL-2 and uses the same signaling pathway^3^, comparing the characteristics of effectors induced by IL-2 and bystander T cells induced by IL-15 would be interesting. Additionally, several studies in mice have proposed that IL-2 depletion is one of the major mechanisms by which Tregs inhibit CD8 + T-cell activation. In a model of experimental autoimmune diabetes established by the transfer of OT-I T cells into transgenic mice expressing OVA in pancreatic β cells, depletion of Tregs led to the expansion of OT-I T cells and their differentiation into a unique subset expressing the NK receptor KLRK1 in addition to effector molecules, including granzyme B and perforin^57^. ScRNA-seq revealed that Treg depletion upregulated the expression of IL-2-responsive genes and IL-2Rα in addition to the expression of NK cell activation receptors and cytotoxic molecules on OT-I T cells. Treatment with IL-2/anti-IL-2 Ab immune complexes (ICs) also augmented the population of KLRK1^+^ CD8 + T cells despite a significant increase in the Treg population. Overall, these results demonstrate that the increase in the abundance of IL-2 induced by exogenous treatments or Treg depletion mediates the differentiation of antigen-specific CD8 + T cells into better effectors in a microenvironment with persistent antigenic stimulation.

In addition to phenotypic and transcriptional changes, IL-2 treatment also significantly altered the epigenetic signature of antigen-specific CD8 + T cells compared to that in untreated mice^48^. The epigenetic signature of antigen-specific CD8 T cells after IL-2 treatment with or without PD-1 blockade became similar to that of effector and memory T cells generated by acute infection instead of exhausted CD8 T cells before or after PD-1 blockade. Notably, the regulatory regions of the *Pdcd1* (encoding PD-1) and *Tox* genes were closed following the administration of IL-2-mediated immunotherapy. Beltra et al. recently reported that the rewiring of epigenetic features and the gain of effector-like characteristics in exhausted CD8 + T cells, driven by IL-2R-mediated signals, depend on Stat5^54^ (Fig. 1e). Stat5 acts as an antagonist of Tox and Tox-mediated exhaustion features and promotes the transition from stem-like T cells to better effectors, thereby fostering effector-like differentiation. These results suggested that the differentiation of stem-like CD8 T cells is flexible and that more functional effector-like CD8 T cells can be generated by IL-2 treatment.

## Historical and current understanding of IL-2 immunotherapy

### IL-2 biology and its shortcomings in cancer immunotherapy

**Fig. 2:**
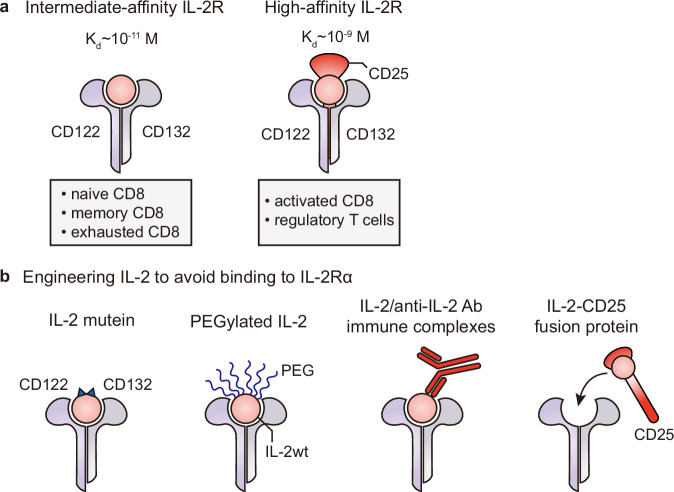
Configuration of IL-2R and engineered IL-2 to avoid binding to IL-2Rα. **a** IL-2R variants include the intermediate-affinity IL-2R, which is composed of CD122 and CD132 and is associated with naive, memory, and exhausted CD8 + T cells. In contrast, the high-affinity IL-2R includes the CD25 subunit alongside CD122 and CD132, which are predominantly found on activated CD8 and regulatory T cells. **b** Engineered IL-2 variants are shown. The panel illustrates the modification of IL-2 to selectively avoid binding to IL-2Rα, thereby enhancing therapeutic efficacy and minimizing side effects. The IL-2 mutein is a mutant version that does not bind to the trimeric high-affinity IL-2R. PEGylated IL-2, which is conjugated with PEG, has a prolonged half-life and decreases the affinity for trimeric IL-2R. IL-2/anti-IL2 antibody immune complexes are designed to increase the half-life and selectively target specific IL-2 receptors. The IL-2-CD25 fusion protein preferentially binds to dimeric intermediate-affinity IL-2R.

IL-2 is a pivotal cytokine the plays a role in the clonal expansion of activated T cells and enhances the cytotoxic activity of CD8 + T cells and NK cells^1,58–60^. IL-2 is mainly produced by activated CD4 + T cells. CD8 + T cells, NK cells, and DCs also produce IL-2 but in limited amounts. IL-2R is composed of three different subunits: IL-2Rα (CD25), IL-2Rβ (CD122), and IL-2Rγ (CD132) (Fig. 2a). IL-2Rβ and IL2Rγ are shared with the IL-15 receptor and play a role in mediating downstream signals^61,62^. Although IL-2Rα does not possess a cytoplasmic moiety for signaling, it presents IL-2 in cis to the IL-2Rβγ complex and increases the binding affinity 10- to 100-fold. Naive T cells usually express dimeric intermediate-affinity receptors consisting of IL-2Rβ and IL-2Rγ. Upon antigenic stimulation, accompanied by IL-2 production, activated T cells upregulate the expression of IL-2α, leading to the generation of trimeric high-affinity receptors of IL-2Rαβγ complexes. Activated T cells utilize secreted IL-2 in an autocrine manner and produce thousands of cells from a single clone. However, Tregs constitutively express IL-2Rα; accordingly, they contain high-affinity IL-2R and preferentially consume IL-2 more readily than naive T cells^63^.

Based on successful preclinical results with IL-2 therapy in cancer models, high-dose IL-2 (aldesleukin; trade name Proleukin) was the first FDA-approved cancer immunotherapeutic drug for treating metastatic RCC in 1992 and metastatic melanoma in 1998, with substantial antitumor efficacy^1,64^. However, due to its short half-life and the prior consumption of IL-2 by Tregs, frequent administration of a high dose of IL-2 is required for clinical benefit, but it also induces life-threatening adverse effects, including vascular leak syndrome (VLS), in addition to various common adverse effects such as central nervous system toxicity, gastrointestinal symptoms (nausea, vomiting, and diarrhea), and hypotension^64,65^. Although earlier studies suggested that VLS is mediated by activated effector cells, including CD4 + T cells, CD8 + T cells, and NK cells^66^, Krieg et al. reported that a fraction of endothelial cells also express IL-2Rα; thus, IL-2 can directly affect these cells, thereby inducing VLS^67^. Furthermore, after the clinical development of IL-2, IL-2 was also found to play a pivotal role in the expansion and maintenance of Treg cells by inhibiting CD8 + T-cell activation in the tumor microenvironment (TME)^68^. Therefore, the clinical application of IL-2 therapy for the treatment of cancer patients is limited due to its toxicity and the activation of Treg cells; ICIs have taken its place as first-line immunotherapy for the treatment of metastatic RCC and melanoma.

### Second generation of IL-2 therapeutics: engineering IL-2 to avoid binding to IL-2Rα

In 2010, Krieg et al. reported that pulmonary edema, one of the major side effects of IL-2, was abolished by using IL-2/anti-IL-2 Ab ICs to direct intermediate-affinity IL-2Rβγ or by eliminating CD25-mediated signals via the use of a blocking Ab or genetic disruption^67^. These findings spurred new avenues for IL-2 immunotherapy, including IL-2 engineering to target particular IL-2R-bearing cell populations, especially IL-2Rβγ-biased IL-2 formulations, to stimulate cytolytic CD8 T cells and NK cells, but not endothelial cells or Tregs, thus preventing off-target effects. There are four major approaches for reducing the binding affinity of IL-2 for CD25: IL-2 muteins, PEGylated IL-2, IL-2/anti-IL-2 Ab ICs, and IL-2-CD25 fusion proteins (Fig. 2b). Here, we provide a brief overview of these approaches, with representative examples in clinical trials, and we refer readers to other recent reviews for more details^4–6^.

IL-2 muteins have amino acid mutations to inhibit their binding to the trimeric high-affinity IL-2R. One type possesses mutations in the binding site of IL-2 to CD25^69^. In another type, the mutated sequences are not directly involved in the binding interface but prevent the binding of the IL-2 mutein to CD25 through conformational changes^70^. Additionally, mutations have been introduced to improve the binding of the IL-2 mutein to CD122. For example, MDNA11 harbors the F42A and E62A mutations to impair CD25 binding and the L80F, R81D, L85V, I86V, and I92F mutations to improve CD122 binding^71^. Roche also constructed an IL-2 variant (IL-2v) harboring F42A, Y45A, and L72G mutations to increase CD122 selectivity. IL-2v was fused to several kinds of mAbs targeting PD-1, carcinoembryonic antigen (CEA), and fibroblast activation protein alpha (FAP) to target PD-1^+^ T cells, tumor cells, and cancer-associated fibroblasts, respectively. These IL-2 muteins were more effective at inducing antitumor activity in preclinical tumor studies by stimulating CD8 + T cells and NK cells than wild-type IL-2 (IL-2wt)^69,70^.

PEGylation has been widely used and clinically proven to extend the half-life of recombinant proteins. Recent advances in technology have allowed for site-specific PEGylation in addition to improvements to pharmacokinetics. NKTR-214 (termed bempegaldesleukin) has an average of six PEG chains on the lysine residues of IL-2^72^. These residues are mostly located at or near the CD25-binding interface, which prevents it from binding to the trimeric high-affinity IL-2R and sustaining binding to the dimeric intermediate-affinity IL-2R. At physiological pH, PEG groups are released from NKTR-214, and IL-2, which has one or two PEG chains, becomes active and stimulates CD8 + T cells and NK cells over Treg cells^73^. Compared with IL-2wt, NKTR-214 was more effective at suppressing the growth of B16F10 tumors, corresponding with an increase in the CD8+/Treg ratio.

Preparing cytokine/anti-cytokine Ab ICs is another approach for overcoming the short half-life of recombinant cytokines^74^. Additionally, IC formation also contributes to its selectivity for cytokine receptors^58^. Boyman et al. presented the differential effects of several mAbs on stimulation by IL-2. The S4B6 anti-IL-2 mAb mediated robust proliferation of CD8 + T cells through the formation of ICs, while IL-2 ICs with JES6-1 selectively expanded Foxp3^+^ Treg cells^75^. S4B6 mAb hinders the binding between IL-2 and CD25 and mediates the conformational changes of IL-2 to promote its binding affinity to dimeric intermediate-affinity IL-2R^76^. In addition to S4B6, other clones, such as JES6-5H4 and MAB602, were identified and exhibited similar mechanisms^67,75–77^. These IL-2 ICs with S4B6, MAB602, or JES6-5H4 exhibited strong antitumor activity in various murine tumor models^77–79^. For human IL-2, an anti-human IL-2 mAb, NARA1, was identified. NARA1 binding to IL-2 allows it to preferentially bind to dimeric IL-2R and exert effective antitumor activity by expanding CD8 + T cells in melanoma models^80,81^. However, in preclinical studies, IL-2 dissociation in the bloodstream from IL-2/NARA1 ICs was observed after in vivo administration, resulting in mitigated effectiveness and a lack of reproducibility. To compensate for these shortcomings, ANV419 (also termed NAR-A1leukin) was developed as a fusion protein of human IL-2 and NARA1^82^. ANV419 mediated effective antitumor responses in several preclinical cancer models, including B16F10 melanoma and LLC lung adenocarcinoma.

Finally, another suggested approach for determining the preference of IL-2 for dimeric intermediate-affinity IL-2R is the prebinding of IL-2 to CD25. However, unlike the strong IL-15/IL-15Rα interaction^83^, the interaction between IL-2 and CD25 is relatively weak, so IL-2 readily dissociates^84^. Moreover, the amino-terminal and carboxy-terminal amino acids of IL-2 are positioned distal to the N-terminus and the IL-2 binding region of CD25, so it is difficult to produce an IL-2/CD25 fusion protein for cis-IL-2/CD25 interactions. To overcome this issue, IL-2 underwent circular permutation and was joined to the extracellular domain of CD25 to create the ALKS 4230 IL-2/CD25 fusion protein (termed Nemvaleukin alfa)^85^. In a preclinical B16 melanoma model, ALKS 4230 was superior to IL-2wt in reducing the likelihood of metastasis and reducing off-target side effects.

### Enhancing therapeutic efficacy by targeting high-affinity IL-2R combined with PD-1 blockade

**Fig. 3:**
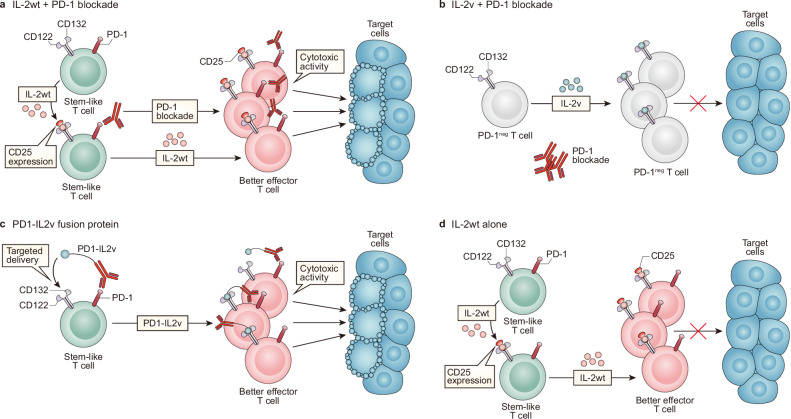
Mode of action of IL-2 derivatives and the requirement for PD-1 blockade in cancer immunotherapy. **a** In IL-2wt + PD-1 blockade, combination therapy with IL-2wt and PD-1-directed immunotherapy leads to an increase in IL-2Rα expression on stem-like T cells and the generation of a large number of better effectors. The addition of anti-PD-1/PD-L1 antibodies further enhances the cytolytic activity of the effectors, thereby effectively suppressing tumor growth. **b** In IL-2v + PD-1 blockade, IL-2v, which is engineered to avoid binding to IL-2Rα, preferentially binds to PD-1^neg^ T cells expressing the IL-2Rβγ dimeric receptor, thereby mediating their expansion. However, since PD-1^neg^ cells, which do not bind to anti-PD-1 antibodies, are mostly tumor nonspecific, IL-2v combined with PD-1 blockade exhibits minimal anticancer efficacy. **c** The PD1-IL2v fusion protein acts as follows: the anti-PD-1 and IL-2v fusion protein delivers IL-2v to PD-1^+^ stem-like T cells, thus enabling the generation of better effectors. Additionally, the anti-PD-1 portion hampers the PD-1/PD-L1 interaction, thus enabling the effectors to exhibit strong cytolytic activity. **d** IL-2wt alone acts as follows: IL-2wt administration upregulated IL-2Rα expression on stem-like T cells, thereby facilitating enhanced binding with IL-2 and the generation of a large number of better effectors. However, because the PD-1/PD-L1 interaction still occurs, this interaction continues to inhibit the cytolytic function of the effectors, leading to continued tumor growth.

Despite the aforementioned efforts and significant antitumor effects reported in preclinical studies, intermediate-affinity IL-2R-targeting engineered IL-2 has achieved disappointing results in the treatment of cancer patients in clinical trials (reviewed in^86^), with the example of negative data from the PIVOT IO-001 phase 3 trial of NKTR-214 by Nektar and Bristol Myers Squibb^87^. These results highlight the need for a new perspective on IL-2 biology and drug design strategies for more promising clinical results. Recent results in chronic viral infection and cancer have shown that strategies targeting dimeric intermediate-affinity IL-2Rs are inadequate for inducing antiviral and antitumor efficacy, and when trimeric high-affinity IL-2Rs are targeted, therapeutic efficacy combined with PD-1 blockade is effectively induced^48,49,52^ (Fig. 3a, b).

A decade ago, the combination of PD-L1 blockade and IL-2 therapy was reported to exhibit a remarkable synergistic effect in terms of decreasing virus titers corresponding to the increased population of virus-specific CD8 + T cells and enhanced effector functions in a model of chronic LCMV infection^47^. Given the minimal expression of CD25 on virus-specific exhausted CD8 + T cells, in the present study, the stimulatory effects of IL-2wt and IL-2v, which are devoid of binding to CD25, were compared^48^. When combined with PD-1 blockade, IL-2v combination therapy did not induce further clonal expansion of virus-specific CD8 + T cells or a decrease in viral titer compared with anti-PD-L1 monotherapy, while IL-2wt combination therapy significantly increased the population of LCMV-specific CD8 + T cells, leading to improved viral control (Fig. 3a, b). Although PD-1 blockade combined with IL-2v significantly increased the population of CD8 T cells, IL-2v selectively expanded PD-1-negative CD8 T cells, which highly express dimeric intermediate-affinity IL-2R (Fig. 3b), indicating inappropriate targeting for immunotherapy. Although virus-specific exhausted CD8 + T cells exhibited limited CD25 expression at the time of administration, IL-2wt augmented CD25 expression, in addition to CD122 and CD132 expression, on proliferating and differentiating virus-specific CD8 + T cells, and these cells became responsive to IL-2 treatment (Fig. 3a). Furthermore, the synergistic effect of IL-2 combined with PD-1 blockade was abolished when cells were treated with blocking anti-CD25 or depleting anti-CD8 Abs. Consistently, in murine tumor models, although IL-2wt expands Tregs more efficiently than IL-2v, IL-2wt exhibited antitumor efficacy superior to that of IL-2v with the clonal expansion of tumor-specific CD8 + T cells and an increased CD8 + T/Treg ratio in the tumor^52^. IFNγ produced by stimulated immune cells may suppress Treg cell expansion in the TME^88^. In contrast, IL-2v leads to the selective expansion of CD39^-^PD-1^-^ T cells. Moreover, an IL-2Rα-biased agonist, which harbors a mutation that reduces its affinity for dimeric IL-2R and does not stimulate resting CD8 + T cells or other CD25^-^ immune cells, has shown a significant synergistic therapeutic effect with anti-PD-1 Abs to suppress the growth of large established tumors^52^. Notably, an IL-2Rα-biased agonist upregulated CD25 expression on tumor-specific T cells, and tumor-specific CD8 + T cells became more susceptible to the agonist via positive feedback and restored the IL-2 signature. Additionally, the therapeutic efficacy of IL-2Rα-biased agonists was nearly abrogated after treatment with CD25-blocking Abs or CD8-depleting Abs. Taken together, these results corroborate that CD25 engagement is essential for the synergy between IL-2 treatment and PD-1 blockade and that CD8 + T cells are major players in mediating antiviral or antitumor effects.

### Exploring PD-1 targeting as an alternative to CD25 engagement in immunotherapy

Intriguingly, a recent report illustrated that the lack of CD25 engagement could be compensated for by a PD-1-targeting strategy, not cancer antigen or TME-targeting strategies^49^ (Fig. 3c). To achieve preferential delivery of IL-2-engineered protein into tumor cells and the TME, researchers have developed CEA-IL2v (termed cergutuzumab amunaleukin)^89^ and FAP-IL2v (termed Simulkafusp alfa)^90^, which are immunocytokines comprising IL-2v and an Ab against CEA and FAP, respectively. In this study, the authors examined the combined effect of FAP-IL2v with PD-1 blockade in a Panc02-H7-Fluc tumor model and observed no therapeutic synergy between them. Given that exhausted CD8 + T cells highly express PD-1, IL-2v was fused with anti-PD-1 antibodies to generate PD1-IL2v. Interestingly, compared with combination therapy with the anti-PD-1 Ab and IL-2v, the PD1-IL2v-fused protein exhibited a superior therapeutic effect on mice bearing Panc02-H7-Fluc, B16-OVA, or MCA-205 tumors. Moreover, when PD1-IL2v was combined with anti-PD-L1 Abs, the PD1-IL2v-fused protein significantly expanded antigen-specific CD8 + T cells and reduced the viral titer in chronic LCMV-infected mice. Similarly, the PD1-IL2v fusion protein also showed a synergistic therapeutic effect with radiation therapy in orthotopic mouse models of KRAS-driven pancreatic ductal adenocarcinoma^53^. Overall, these results demonstrated that PD-1-targeted delivery of IL-2v leads to the clonal expansion of antigen-specific exhausted CD8 + T cells through appropriate targeting strategies, thereby enhancing the effectiveness of immunotherapies.

Interestingly, IL-2-mediated signaling, rather than PD-1 blockade, results in the differentiation of the stem-like CD8 T-cell subset into better effectors. However, the generation of better effectors and their clonal expansion were not enough to mediate the antiviral effect (Fig. 3d). PD-1 blockade following IL-2 treatment is required for lowering virus titers in chronically infected mice (Fig. 3a), suggesting that IL-2 and PD-1 blockade in combination therapy and the PD1-IL2v fusion protein have different roles: IL-2 reprograms the differentiation of stem-like CD8 T cells into better effectors and leads to clonal expansion, while PD-1 blockade boosts the killing activity of expanded antigen-specific CD8 T cells by inhibiting the PD-1/PD-L1 interaction between CD8 T cells and target cells^48^ (Fig. 3a).

## Concluding remarks

We reviewed the evolving understanding of IL-2 biology in the context of immunotherapy, in line with current trends, and summarized recent findings on the impact of IL-2-mediated immunotherapy on the differentiation of exhausted CD8 + T cells. Various efforts have been made to engineer IL-2 to avoid severe side effects and off-target effects caused by IL-2wt. Most of these initiatives have focused on reducing binding to IL-2Rα. Recent findings have underscored the significance of engaging CD25 or targeting antigen-specific CD8 T cells for the therapeutic efficacy of IL-2-mediated therapies, thereby opening new potential avenues for combination therapies with current immune checkpoint treatments. PD1-IL2v is a promising approach for stimulating antigen-specific CD8 + T cells while minimizing off-target side effects. However, as seen in previous clinical studies involving engineered IL-2, encouraging results from preclinical studies do not always translate to positive outcomes in clinical trials. Despite this finding, as advancements continue in this field, we anticipate that new agents derived from our evolving understanding will find a significant place in the future landscape of cancer immunotherapy.

## References

[1] Spolski, R., Li, P. & Leonard, W. J. Biology and regulation of IL-2: from molecular mechanisms to human therapy. *Nat. Rev. Immunol.* **18**, 648–659 (2018). 30089912 10.1038/s41577-018-0046-y

[2] Gaffen, S. L. & Liu, K. D. Overview of interleukin-2 function, production and clinical applications. *Cytokine* **28**, 109–123 (2004). 15473953 10.1016/j.cyto.2004.06.010

[3] Wolfarth, A. A. et al. Advancements of common gamma-chain family cytokines in cancer immunotherapy. *Immune Netw.* **22**, e5 (2022). 35291658 10.4110/in.2022.22.e5 PMC8901704

[4] Hernandez, R., Poder, J., LaPorte, K. M. & Malek, T. R. Engineering IL-2 for immunotherapy of autoimmunity and cancer. *Nat. Rev. Immunol.* **22**, 614–628 (2022). 35217787 10.1038/s41577-022-00680-w

[5] Mullard, A. Restoring IL-2 to its cancer immunotherapy glory. *Nat. Rev. Drug Discov.* **20**, 163–165 (2021). 33603157 10.1038/d41573-021-00034-6

[6] Raeber, M. E., Sahin, D. & Boyman, O. Interleukin-2-based therapies in cancer. *Sci. Transl. Med.* **14**, eabo5409 (2022). 36350987 10.1126/scitranslmed.abo5409

[7] Lee, J. B., Kim, H. R. & Ha, S. J. Immune checkpoint inhibitors in 10 years: contribution of basic research and clinical application in cancer immunotherapy. *Immune Netw.* **22**, e2 (2022). 35291660 10.4110/in.2022.22.e2 PMC8901707

[8] Wherry, E. J., Blattman, J. N., Murali-Krishna, K., van der Most, R. & Ahmed, R. Viral persistence alters CD8 T-cell immunodominance and tissue distribution and results in distinct stages of functional impairment. *J. Virol.* **77**, 4911–4927 (2003). 12663797 10.1128/JVI.77.8.4911-4927.2003 PMC152117

[9] Chikuma, S. et al. PD-1-mediated suppression of IL-2 production induces CD8+ T cell anergy in vivo. *J. Immunol.* **182**, 6682–6689 (2009). 19454662 10.4049/jimmunol.0900080

[10] Liu, R. et al. PD-1 signaling negatively regulates the common cytokine receptor gamma chain via MARCH5-mediated ubiquitination and degradation to suppress anti-tumor immunity. *Cell Res.* **33**, 923–939 (2023). 37932447 10.1038/s41422-023-00890-4 PMC10709454

[11] Spranger, S. et al. Mechanism of tumor rejection with doublets of CTLA-4, PD-1/PD-L1, or IDO blockade involves restored IL-2 production and proliferation of CD8(+) T cells directly within the tumor microenvironment. *J. Immunother. Cancer* **2**, 3 (2014). 24829760 10.1186/2051-1426-2-3 PMC4019906

[12] Hashimoto, M. et al. CD8 T cell exhaustion in chronic infection and cancer: opportunities for interventions. *Annu. Rev. Med.* **69**, 301–318 (2018). 29414259 10.1146/annurev-med-012017-043208

[13] McLane, L. M., Abdel-Hakeem, M. S. & Wherry, E. J. CD8 T cell exhaustion during chronic viral infection and cancer. *Annu. Rev. Immunol.* **37**, 457–495 (2019). 30676822 10.1146/annurev-immunol-041015-055318

[14] Zajac, A. J. et al. Viral immune evasion due to persistence of activated T cells without effector function. *J. Exp. Med.* **188**, 2205–2213 (1998). 9858507 10.1084/jem.188.12.2205 PMC2212420

[15] Barber, D. L. et al. Restoring function in exhausted CD8 T cells during chronic viral infection. *Nature* **439**, 682–687 (2006). 16382236 10.1038/nature04444

[16] Im, S. J. et al. Defining CD8+ T cells that provide the proliferative burst after PD-1 therapy. *Nature* **537**, 417–421 (2016). 27501248 10.1038/nature19330 PMC5297183

[17] He, R. et al. Follicular CXCR5- expressing CD8(+) T cells curtail chronic viral infection. *Nature* **537**, 412–428 (2016). 27501245 10.1038/nature19317

[18] Utzschneider, D. T. et al. T Cell factor 1-expressing memory-like CD8(+) T cells sustain the immune response to chronic viral infections. *Immunity* **45**, 415–427 (2016). 27533016 10.1016/j.immuni.2016.07.021

[19] Siddiqui, I. et al. Intratumoral Tcf1(+)PD-1(+)CD8(+) T cells with stem-like properties promote tumor control in response to vaccination and checkpoint blockade immunotherapy. *Immunity* **50**, 195–211.e110 (2019). 30635237 10.1016/j.immuni.2018.12.021

[20] Miller, B. C. et al. Subsets of exhausted CD8(+) T cells differentially mediate tumor control and respond to checkpoint blockade. *Nat. Immunol.* **20**, 326–336 (2019). 30778252 10.1038/s41590-019-0312-6 PMC6673650

[21] Eberhardt, C. S. et al. Functional HPV-specific PD-1(+) stem-like CD8 T cells in head and neck cancer. *Nature* **597**, 279–284 (2021). 34471285 10.1038/s41586-021-03862-z PMC10201342

[22] Hudson, W. H. et al. Proliferating transitory T cells with an effector-like transcriptional signature emerge from PD-1(+) Stem-like CD8(+) T cells during chronic infection. *Immunity* **51**, 1043–1058.e1044 (2019). 31810882 10.1016/j.immuni.2019.11.002 PMC6920571

[23] Im, S. J. & Ha, S. J. Re-defining T-cell exhaustion: subset, function, and regulation. *Immune Netw.* **20**, e2 (2020). 32158590 10.4110/in.2020.20.e2 PMC7049579

[24] Utzschneider, D. T. et al. Early precursor T cells establish and propagate T cell exhaustion in chronic infection. *Nat. Immunol.* **21**, 1256–1266 (2020). 32839610 10.1038/s41590-020-0760-z

[25] Yao, C. et al. BACH2 enforces the transcriptional and epigenetic programs of stem-like CD8(+) T cells. *Nat. Immunol.* **22**, 370–380 (2021). 33574619 10.1038/s41590-021-00868-7 PMC7906956

[26] Wu, T. et al. The TCF1-Bcl6 axis counteracts type I interferon to repress exhaustion and maintain T cell stemness. *Sci. Immunol.* **1**, eaai8593 (2016). 28018990 10.1126/sciimmunol.aai8593 PMC5179228

[27] Tsui, C. et al. MYB orchestrates T cell exhaustion and response to checkpoint inhibition. *Nature* **609**, 354–360 (2022). 35978192 10.1038/s41586-022-05105-1 PMC9452299

[28] Gautam, S. et al. The transcription factor c-Myb regulates CD8(+) T cell stemness and antitumor immunity. *Nat. Immunol.* **20**, 337–349 (2019). 30778251 10.1038/s41590-018-0311-z PMC6489499

[29] Alfei, F. et al. TOX reinforces the phenotype and longevity of exhausted T cells in chronic viral infection. *Nature* **571**, 265–269 (2019). 31207605 10.1038/s41586-019-1326-9

[30] Khan, O. et al. TOX transcriptionally and epigenetically programs CD8(+) T cell exhaustion. *Nature* **571**, 211–218 (2019). 31207603 10.1038/s41586-019-1325-x PMC6713202

[31] Scott, A. C. et al. TOX is a critical regulator of tumour-specific T cell differentiation. *Nature* **571**, 270–274 (2019). 31207604 10.1038/s41586-019-1324-y PMC7698992

[32] Shin, B. et al. Effector CD4 T cells with progenitor potential mediate chronic intestinal inflammation. *J. Exp. Med* **215**, 1803–1812 (2018). 29915024 10.1084/jem.20172335 PMC6028516

[33] Karmaus, P. W. F. et al. Metabolic heterogeneity underlies reciprocal fates of T(H)17 cell stemness and plasticity. *Nature* **565**, 101–105 (2019). 30568299 10.1038/s41586-018-0806-7 PMC6420879

[34] Schnell, A. et al. Stem-like intestinal Th17 cells give rise to pathogenic effector T cells during autoimmunity. *Cell* **184**, 6281–6298.e6223 (2021). 34875227 10.1016/j.cell.2021.11.018 PMC8900676

[35] Lee, S. et al. Defining a TCF1-expressing progenitor allogeneic CD8(+) T cell subset in acute graft-versus-host disease. *Nat. Commun.* **14**, 5869 (2023). 37737221 10.1038/s41467-023-41357-9 PMC10516895

[36] Im, S. J. et al. Characteristics and anatomic location of PD-1(+)TCF1(+) stem-like CD8 T cells in chronic viral infection and cancer. *Proc. Natl Acad. Sci. USA* **120**, e2221985120 (2023). 37782797 10.1073/pnas.2221985120 PMC10576122

[37] Mueller, S. N. et al. PD-L1 has distinct functions in hematopoietic and nonhematopoietic cells in regulating T cell responses during chronic infection in mice. *J. Clin. Investig.* **120**, 2508–2515 (2010). 20551512 10.1172/JCI40040 PMC2898584

[38] Crozat, K. et al. Cutting edge: expression of XCR1 defines mouse lymphoid-tissue resident and migratory dendritic cells of the CD8alpha+ type. *J. Immunol.* **187**, 4411–4415 (2011). 21948982 10.4049/jimmunol.1101717

[39] Dahling, S. et al. Type 1 conventional dendritic cells maintain and guide the differentiation of precursors of exhausted T cells in distinct cellular niches. *Immunity* **55**, 656–670.e658 (2022). 35366396 10.1016/j.immuni.2022.03.006

[40] Jansen, C. S. et al. An intra-tumoral niche maintains and differentiates stem-like CD8 T cells. *Nature* **576**, 465–470 (2019). 31827286 10.1038/s41586-019-1836-5 PMC7108171

[41] Magen, A. et al. Intratumoral dendritic cell-CD4(+) T helper cell niches enable CD8(+) T cell differentiation following PD-1 blockade in hepatocellular carcinoma. *Nat. Med.* **29**, 1389–1399 (2023). 37322116 10.1038/s41591-023-02345-0 PMC11027932

[42] Im, S. J., Konieczny, B. T., Hudson, W. H., Masopust, D. & Ahmed, R. PD-1+ stemlike CD8 T cells are resident in lymphoid tissues during persistent LCMV infection. *Proc. Natl Acad. Sci. USA* **117**, 4292–4299 (2020). 32034098 10.1073/pnas.1917298117 PMC7049149

[43] Gill, A. L. et al. PD-1 blockade increases the self-renewal of stem-like CD8 T cells to compensate for their accelerated differentiation into effectors. *Sci. Immunol.* **8**, eadg0539 (2023). 37624909 10.1126/sciimmunol.adg0539 PMC10798572

[44] Sade-Feldman, M. et al. Defining T cell states associated with response to checkpoint immunotherapy in melanoma. *Cell* **175**, 998–1013.e1020 (2018). 30388456 10.1016/j.cell.2018.10.038 PMC6641984

[45] Koh, J. et al. TCF1(+)PD-1(+) tumour-infiltrating lymphocytes predict a favorable response and prolonged survival after immune checkpoint inhibitor therapy for non-small-cell lung cancer. *Eur. J. Cancer* **174**, 10–20 (2022). 35970031 10.1016/j.ejca.2022.07.004

[46] Wang, X. Q. et al. Spatial predictors of immunotherapy response in triple-negative breast cancer. *Nature* **621**, 868–876 (2023). 37674077 10.1038/s41586-023-06498-3 PMC10533410

[47] West, E. E. et al. PD-L1 blockade synergizes with IL-2 therapy in reinvigorating exhausted T cells. *J. Clin. Investig.* **123**, 2604–2615 (2013). 23676462 10.1172/JCI67008 PMC3668811

[48] Hashimoto, M. et al. PD-1 combination therapy with IL-2 modifies CD8(+) T cell exhaustion program. *Nature* **610**, 173–181 (2022). 36171288 10.1038/s41586-022-05257-0 PMC9793890

[49] Codarri Deak, L. et al. PD-1-cis IL-2R agonism yields better effectors from stem-like CD8(+) T cells. *Nature* **610**, 161–172 (2022). 36171284 10.1038/s41586-022-05192-0 PMC9534752

[50] Raju, S., Verbaro, D. J. & Egawa, T. PD-1 signaling promotes control of chronic viral infection by restricting type-I-interferon-mediated tissue damage. *Cell Rep.* **29**, 2556–2564.e2553 (2019). 31775026 10.1016/j.celrep.2019.10.092 PMC6894421

[51] Abdel-Hakeem, M. S. et al. Epigenetic scarring of exhausted T cells hinders memory differentiation upon eliminating chronic antigenic stimulation. *Nat. Immunol.* **22**, 1008–1019 (2021). 34312545 10.1038/s41590-021-00975-5 PMC8323971

[52] Wu, W. et al. IL-2Ralpha-biased agonist enhances antitumor immunity by invigorating tumor-infiltrating CD25(+)CD8(+) T cells. *Nat. Cancer* **4**, 1309–1325 (2023). 37550516 10.1038/s43018-023-00612-0

[53] Piper, M. et al. Simultaneous targeting of PD-1 and IL-2Rbetagamma with radiation therapy inhibits pancreatic cancer growth and metastasis. *Cancer Cell* **41**, 950–969.e956 (2023). 37116489 10.1016/j.ccell.2023.04.001 PMC10246400

[54] Beltra, J. C. et al. Stat5 opposes the transcription factor Tox and rewires exhausted CD8(+) T cells toward durable effector-like states during chronic antigen exposure. *Immunity* **56**, 2699–2718.e2611 (2023). 38091951 10.1016/j.immuni.2023.11.005 PMC10752292

[55] Paprckova, D., Salyova, E., Michalik, J. & Stepanek, O. Bystander activation in memory and antigen-inexperienced memory-like CD8 T cells. *Curr. Opin. Immunol.* **82**, 102299 (2023). 36913776 10.1016/j.coi.2023.102299

[56] Lee, H. G., Cho, M. J. & Choi, J. M. Bystander CD4(+) T cells: crossroads between innate and adaptive immunity. *Exp. Mol. Med.* **52**, 1255–1263 (2020). 32859954 10.1038/s12276-020-00486-7 PMC8080565

[57] Tsyklauri, O. et al. Regulatory T cells suppress the formation of potent KLRK1 and IL-7R expressing effector CD8 T cells by limiting IL-2. *Elife* **12**, e79342 (2023). 36705564 10.7554/eLife.79342 PMC9977273

[58] Boyman, O. & Sprent, J. The role of interleukin-2 during homeostasis and activation of the immune system. *Nat. Rev. Immunol.* **12**, 180–190 (2012). 22343569 10.1038/nri3156

[59] Ross, S. H. & Cantrell, D. A. Signaling and function of interleukin-2 in T lymphocytes. *Annu. Rev. Immunol.* **36**, 411–433 (2018). 29677473 10.1146/annurev-immunol-042617-053352 PMC6472684

[60] Malek, T. R. The biology of interleukin-2. *Annu. Rev. Immunol.* **26**, 453–479 (2008). 18062768 10.1146/annurev.immunol.26.021607.090357

[61] Nakamura, Y. et al. Heterodimerization of the IL-2 receptor beta- and gamma-chain cytoplasmic domains is required for signalling. *Nature* **369**, 330–333 (1994). 8183373 10.1038/369330a0

[62] Nelson, B. H., Lord, J. D. & Greenberg, P. D. Cytoplasmic domains of the interleukin-2 receptor beta and gamma chains mediate the signal for T-cell proliferation. *Nature* **369**, 333–336 (1994). 7514277 10.1038/369333a0

[63] Malek, T. R. & Castro, I. Interleukin-2 receptor signaling: at the interface between tolerance and immunity. *Immunity* **33**, 153–165 (2010). 20732639 10.1016/j.immuni.2010.08.004 PMC2946796

[64] Jiang, T., Zhou, C. & Ren, S. Role of IL-2 in cancer immunotherapy. *Oncoimmunology* **5**, e1163462 (2016). 27471638 10.1080/2162402X.2016.1163462 PMC4938354

[65] Skrombolas, D. & Frelinger, J. G. Challenges and developing solutions for increasing the benefits of IL-2 treatment in tumor therapy. *Expert Rev. Clin. Immunol.* **10**, 207–217 (2014). 24410537 10.1586/1744666X.2014.875856 PMC4072333

[66] Cotran, R. S. et al. Endothelial activation during interleukin 2 immunotherapy. A possible mechanism for the vascular leak syndrome. *J. Immunol.* **140**, 1883–1888 (1988). 3279124

[67] Krieg, C., Letourneau, S., Pantaleo, G. & Boyman, O. Improved IL-2 immunotherapy by selective stimulation of IL-2 receptors on lymphocytes and endothelial cells. *Proc. Natl Acad. Sci. USA* **107**, 11906–11911 (2010). 20547866 10.1073/pnas.1002569107 PMC2900642

[68] Mitra, S. & Leonard, W. J. Biology of IL-2 and its therapeutic modulation: mechanisms and strategies. *J. Leukoc. Biol.* **103**, 643–655 (2018). 29522246 10.1002/JLB.2RI0717-278R

[69] Carmenate, T. et al. Human IL-2 mutein with higher antitumor efficacy than wild type IL-2. *J. Immunol.* **190**, 6230–6238 (2013). 23677467 10.4049/jimmunol.1201895

[70] Levin, A. M. et al. Exploiting a natural conformational switch to engineer an interleukin-2 ‘superkine. *Nature* **484**, 529–533 (2012). 22446627 10.1038/nature10975 PMC3338870

[71] Merchant, R. et al. Fine-tuned long-acting interleukin-2 superkine potentiates durable immune responses in mice and non-human primate. *J. Immunother. Cancer* **10**, e003155 (2022). 35058325 10.1136/jitc-2021-003155 PMC8772458

[72] Charych, D. H. et al. NKTR-214, an engineered cytokine with biased il2 receptor binding, increased tumor exposure, and marked efficacy in mouse tumor models. *Clin. Cancer Res* **22**, 680–690 (2016). 26832745 10.1158/1078-0432.CCR-15-1631

[73] Charych, D. et al. Modeling the receptor pharmacology, pharmacokinetics, and pharmacodynamics of NKTR-214, a kinetically-controlled interleukin-2 (IL2) receptor agonist for cancer immunotherapy. *PLoS One* **12**, e0179431 (2017). 28678791 10.1371/journal.pone.0179431 PMC5497954

[74] Finkelman, F. D. et al. Anti-cytokine antibodies as carrier proteins. Prolongation of in vivo effects of exogenous cytokines by injection of cytokine-anti-cytokine antibody complexes. *J. Immunol.* **151**, 1235–1244 (1993). 8393043

[75] Boyman, O., Kovar, M., Rubinstein, M. P., Surh, C. D. & Sprent, J. Selective stimulation of T cell subsets with antibody-cytokine immune complexes. *Science* **311**, 1924–1927 (2006). 16484453 10.1126/science.1122927

[76] Spangler, J. B. et al. Antibodies to interleukin-2 elicit selective T cell subset potentiation through distinct conformational mechanisms. *Immunity* **42**, 815–825 (2015). 25992858 10.1016/j.immuni.2015.04.015 PMC4439582

[77] Tomala, J., Chmelova, H., Mrkvan, T., Rihova, B. & Kovar, M. In vivo expansion of activated naive CD8+ T cells and NK cells driven by complexes of IL-2 and anti-IL-2 monoclonal antibody as novel approach of cancer immunotherapy. *J. Immunol.* **183**, 4904–4912 (2009). 19801515 10.4049/jimmunol.0900284

[78] Arenas-Ramirez, N., Woytschak, J. & Boyman, O. Interleukin-2: biology, design and application. *Trends Immunol.* **36**, 763–777 (2015). 26572555 10.1016/j.it.2015.10.003

[79] Reyes, R. M. et al. CD122-directed interleukin-2 treatment mechanisms in bladder cancer differ from alphaPD-L1 and include tissue-selective gammadelta T cell activation. *J. Immunother. Cancer* **9**, e002051 (2021). 33849925 10.1136/jitc-2020-002051 PMC8051418

[80] Arenas-Ramirez, N. et al. Improved cancer immunotherapy by a CD25-mimobody conferring selectivity to human interleukin-2. *Sci. Transl. Med.* **8**, 367ra166 (2016). 27903862 10.1126/scitranslmed.aag3187

[81] Raeber, M. E., Rosalia, R. A., Schmid, D., Karakus, U. & Boyman, O. Interleukin-2 signals converge in a lymphoid-dendritic cell pathway that promotes anticancer immunity. *Sci. Transl. Med.* **12**, eaba5464 (2020). 32938795 10.1126/scitranslmed.aba5464

[82] Sahin, D. et al. An IL-2-grafted antibody immunotherapy with potent efficacy against metastatic cancer. *Nat. Commun.* **11**, 6440 (2020). 33353953 10.1038/s41467-020-20220-1 PMC7755894

[83] Stonier, S. W. & Schluns, K. S. Trans-presentation: a novel mechanism regulating IL-15 delivery and responses. *Immunol. Lett.* **127**, 85–92 (2010). 19818367 10.1016/j.imlet.2009.09.009 PMC2808451

[84] Rickert, M., Wang, X., Boulanger, M. J., Goriatcheva, N. & Garcia, K. C. The structure of interleukin-2 complexed with its alpha receptor. *Science* **308**, 1477–1480 (2005). 15933202 10.1126/science.1109745

[85] Lopes, J. E. et al. ALKS 4230: a novel engineered IL-2 fusion protein with an improved cellular selectivity profile for cancer immunotherapy. *J. Immunother. Cancer* **8**, e000673 (2020). 32317293 10.1136/jitc-2020-000673 PMC7204809

[86] Raeber, M. E., Sahin, D., Karakus, U. & Boyman, O. A systematic review of interleukin-2-based immunotherapies in clinical trials for cancer and autoimmune diseases. *EBioMedicine* **90**, 104539 (2023). 37004361 10.1016/j.ebiom.2023.104539 PMC10111960

[87] American Association for Cancer Research. Bempeg failure unlikely to affect other IL2 drugs. *Cancer Discov.* **12**, 160–1605 (2022). 10.1158/2159-8290.CD-NB2022-0036 35553619

[88] Overacre-Delgoffe, A. E. et al. Interferon-gamma drives T(reg) fragility to promote anti-tumor immunity. *Cell* **169**, 1130–1141.e1111 (2017). 28552348 10.1016/j.cell.2017.05.005 PMC5509332

[89] Klein, C. et al. Cergutuzumab amunaleukin (CEA-IL2v), a CEA-targeted IL-2 variant-based immunocytokine for combination cancer immunotherapy: overcoming limitations of aldesleukin and conventional IL-2-based immunocytokines. *Oncoimmunology* **6**, e1277306 (2017). 28405498 10.1080/2162402X.2016.1277306 PMC5384349

[90] Waldhauer, I. et al. Simlukafusp alfa (FAP-IL2v) immunocytokine is a versatile combination partner for cancer immunotherapy. *MAbs* **13**, 1913791 (2021). 33974508 10.1080/19420862.2021.1913791 PMC8115765

